# Evaluation of In Vitro-Derived Hop Plantlets, cv. Columbus and Magnum, as Potential Source of Bioactive Compounds

**DOI:** 10.3390/antiox13080909

**Published:** 2024-07-28

**Authors:** Leandra Leto, Claudia Favari, Anna Agosti, Lorenzo Del Vecchio, Andrea Di Fazio, Letizia Bresciani, Pedro Mena, Valeria Guarrasi, Martina Cirlini, Benedetta Chiancone

**Affiliations:** 1 Department of Food and Drug, University of Parma, Viale Parco Area delle Scienze 27/A, 43124 Parma, Italy; leandra.leto@unipr.it (L.L.); claudia.favari@unipr.it (C.F.); anna.agosti@unipr.it (A.A.); lorenzo.delvecchio@unipr.it (L.D.V.); andrea.difazio@studenti.unipr.it (A.D.F.); letizia.bresciani@unipr.it (L.B.); pedro.mena@unipr.it (P.M.); martina.cirlini@unipr.it (M.C.); 2 Institute of Biophysics, National Research Council (CNR), Via Ugo La Malfa 153, 90146 Palermo, Italy; valeria.guarrasi@ibf.cnr.it

**Keywords:** antioxidant activity, *Humulus lupulus* L., micropropagation, molecular profile, (poly)phenolic content, secondary metabolites

## Abstract

The demand for bioactive secondary metabolites of natural origin is increasing every day. Micropropagation could be a strategy to respond more quickly to market demands, regardless of seasonality. This research aims to evaluate in vitro-grown plants of two hop varieties, namely Columbus and Magnum, as a potential source of bioactive compounds. The extracts were characterized in terms of total phenolic content by a Folin–Ciocalteu assay and antioxidant capacity by DPPH^•^, ABTS^+^, and FRAP assays. The bioactive compound profile of the extracts from both varieties was determined by using UPLC-ESI-QqQ-MS/MS. The results confirmed richness in (poly)phenols and other secondary metabolites of the in vitro-grown hop plantlets. Thirty-two compounds belonging to the major families of phytochemicals characteristic of the species were identified, and twenty-six were quantified, mainly flavonoids, including xanthohumol and isoxanthohumol, phenolic acids, as well as α- and β-acids. This study confirms the validity of in vitro-derived hop plantlets as source of bioactive compounds to be used in the nutraceutical, pharmaceutical, and food industries.

## 1. Introduction

Nowadays, interest in products of natural origin is increasing, both among conscious consumers and in the production world. For this reason, there is a growing interest in expanding the number of plant species that can be considered as a source of compounds of interest, and several research efforts are aimed at characterizing and evaluating new matrices that can be used as bio-factories in the future [1]. *Humulus lupulus* L., hop, is a plant that is cultivated all over the world. In recent years, cultivation has also resumed in Italy, where the number of cultivated varieties is increasing and spreading across the peninsula, from the North to the South [2,3,4]. Hop is a very well-known plant due to the indispensable role of its cones in beer production. Recently, thanks to circular economy approaches, vegetative biomass, usually considered as waste, has also been studied to evaluate its alternative uses in the production of compost [2].

In plants, in addition to primary metabolism, which is capable of producing molecules that are important for the survival of the plant itself, secondary metabolism takes place under certain conditions. Indeed, due to abiotic or biotic threats, plants lose their homeostasis and activate other biosynthetic pathways, collectively referred to as secondary metabolism [2,4,5]. The products of secondary metabolism in plants mainly have the function of defense, competition, or attraction of pollinating insects. The biologically active molecules (also called bioactive substances or biologically active plant compounds) with low molecular weight found in hop cones belong to different chemical classes such as (poly)phenols (xanthohumol, (epi)catechins, quercetin and its glycoside derivatives, caffeoylquinic acids, sinapic acid, etc.), α- and β-bitter acids (humulone, cohumulone, lupulone, and colupulone), and terpenoids (β-myrcene, caryophyllene, humulene, β-farnesene, and α- and β-selinene) [6,7,8,9,10].

One of the most important compounds characteristic of hop cones, also present in the vegetative biomass, is xanthohumol. In the last decades, a few in vitro and animal studies have shown several potentially beneficial actions of xanthohumol, mainly against cancer development [11,12], but also on body weight and cardiometabolic risk factors, as recently reviewed by Neumann et al. [13]. However, further evidence from human studies is needed to confirm these potential health-related benefits of xanthohumol, as well as studies that comprehensively evaluate its metabolism in the human body. Another use of xanthohumol could be the management of primary insomnia. It has been reported that this phytochemical in combination with other herbs, such as valerian, could improve the quality of night sleep [14], although additional research is necessary to validate this potential beneficial effect.

Recent studies [4,15] have demonstrated that potentially bioactive compounds, characteristic of hop plants, are also present in in vitro-derived plantlets. Recovering these highly requested compounds from plantlets would help overcome some of the problems closely linked to the high susceptibility of their secondary metabolism to environmental growing conditions [5,16]. In fact, when grown in vitro, plantlets are assured to be under controlled conditions, thus, the type and concentration of bioactive compounds can be standardized in order to guarantee a homogeneous product to stakeholders. Moreover, since hop is a deciduous plant that loses its leaves at the end of summer, there could be problems in the vegetative biomass supply throughout the year, while resorting to micropropagated plants, whose growing is independent of seasonality, would also solve this problem.

Therefore, in vitro cultures are an additional resource alongside plants grown in open fields for nurseries, companies, and research, thanks to their standardized growing conditions and their production being independent of seasonality. 

This research aims at evaluating in vitro-derived hop plantlets, cv. Columbus and Magnum, as a source of bioactive compounds, setting up, firstly, an efficient extraction protocol, and secondly, characterizing them in terms of antioxidant activity and phytochemical profile.

## 2. Materials and Methods

### 2.1. Chemicals and Solvents

All chemicals and solvents used in this study were of analytical grade. HPLC-grade solvents and reagents were purchased from VWR International (Radnor, PA, USA). 3,4-dihydroxybenzoic acid (protocatechuic acid), 3,4,5-trihydroxybenzoic acid (gallic acid), 3-caffeoylquinic acid, 4-caffeoylquinic acid, 5-caffeoylquinic acid, quercetin-3-*O*-glucoside, xanthohumol, isoxanthohumol, and 8-prenylnaringenin were purchased from Merck (Darmstadt, Germany). Humulone, isohumulone, and sinapic acid acyl- glucoside were purchased from Toronto Research Chemicals (Toronto, ON, Canada), and kaempferol-3-*O*-rutinoside and quercetin-7-*O*-glucoside were purchased from ExtraSynthese (Genay, France). (+)-Catechin was purchased from MedChemExpress (Monmouth Junction, NJ, USA), and (−)-epicatechin and 6-prenylnaringenin were purchased from Biosynth Ltd. (Compton, UK).

### 2.2. Plantlet Materials

In vitro-derived hop plantlets used as a matrix for bioactive compound extraction belonged to the cultivars Columbus and Magnum. These two cultivars were selected and developed in the 1980s by Professor Zimmerman for Hop union Inc. Together with Tomahawk and Zeus, Columbus is one of the CTZ hops (Columbus, Tomahawk, and Zeus; three varieties of hops indicated on the market with this acronym as they have very similar characteristics). Columbus has an α-acid content of 14–16%, making it an ideal cultivar for bittering beers such as Pale Ales, India Pale Ales, Stouts, and other Imperial styles. Magnum, released in 1993, is a hybrid of American Galena, obtained at Hüll Hop Research Centre, characterized by an α-acid content of 12–13%, that makes it one of the most appreciated varieties among bitter hops.

To obtain the plant material to be used as a matrix for bioactive compound extraction, ten uninodal microcuttings of both cultivars were grown in 500 mL glass jars. Each jar contained 100 mL of growing medium composed of Murashige and Skoog (MS) [17] salt and vitamin mixture (1×), 30 g/L sucrose, and 8.0 g/L plant agar, with the pH adjusted to 5.8. Jars with the culture medium underwent a sterilization cycle in an autoclave for 20 min at 121 °C. The cultures were maintained in a growth chamber at 25 ± 1 °C with a light intensity of 20 μmol m^−2^ s^−1^ under a 16 h photoperiod.

### 2.3. Sample Extraction

**Figure 1:**
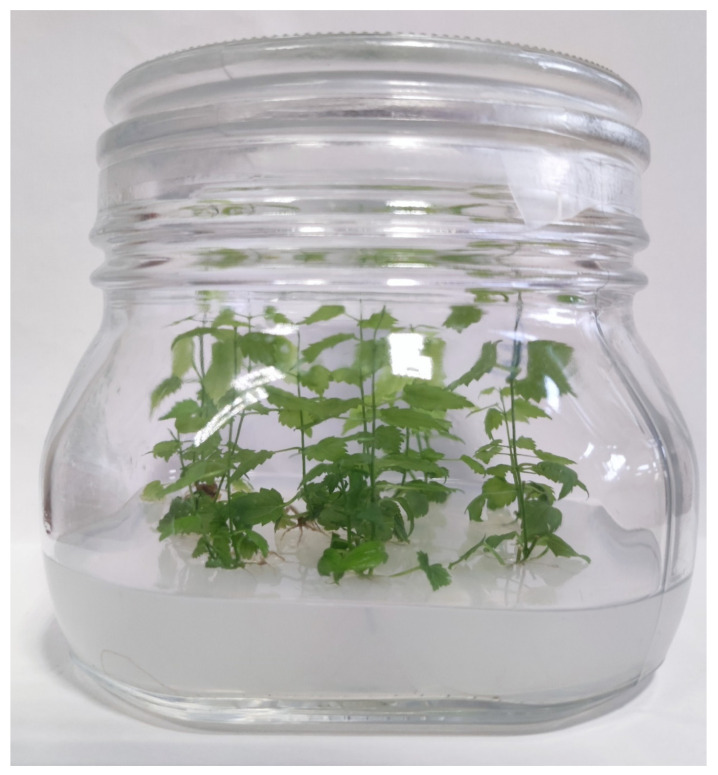
Columbus plantlets cultured in vitro for five weeks.

After five weeks, the in vitro microcuttings developed into plantlets with shoots and roots (Figure 1).

Before the extraction step, the plantlets were washed with distilled water to remove any residues of agar and then weighed and freeze-dried with a Lio-5P lyophilizer (5Pascal, Milan, Italy). The lyophilized material was then powdered using a pestle and mortar. The resulting powder (0.5 g) was suspended in 10 mL of an 80/20 ethanol/water solution (*v*/*v*). To establish a robust protocol, the extraction was conducted using two techniques: the suspension was placed on a shaker for 2 h at 200 strokes/minute at room temperature on a digital agitator (HS 501, IKA-Werke GmbH & Co, Staufen, Germany), or inside an ultrasonic sonication bath for 30 min at 25 °C (VWR International, Milan, Italy) [18,19]. The conditions described above were set based on experimental tests conducted with the aim of causing no change in the temperature of the ultrasonic bath during the extraction time; in particular, the temperature was monitored using a thermometer. To separate the extract from the solid fraction, a centrifuge (Centrifuge 4206, Alc International, Pévy, France) was used at 5000 rpm for 10 min at room temperature. The supernatants were recovered and further diluted (1/5 ratio with 80/20 ethanol/water mixture) before proceeding with spectrophotometric tests, as described in a previous study [4]. Each extraction procedure was repeated in duplicate for each sample.

### 2.4. Determination of Total Phenolic Content and Antioxidant Activity of Extracts

The determination of total phenolic content (TPC) and antioxidant activity (AO) of sample extracts was carried out using different spectrophotometric tests, following protocols described by Chiancone et al. [4]. The Folin–Ciocalteu test was employed for TPC determination, while three assays were used for AO estimation: 2,2-diphenyl-1-picrylhydrazyl (DPPH^•^) and 2,2′-azinobis (3-ethylbenzothiazoline-6-sulfonic acid) (ABTS^+^) assays to assess the radical scavenging activity of the sample extracts, and a ferric reducing antioxidant power (FRAP) assay to determine the reducing capacity. Gallic acid was used as a reference compound for TPC estimation, allowing the expression of data as mg/g GAE (Gallic Acid Equivalents), while 6-hydroxy-2,5,7,8-tetramethylchroman-2-carboxylic acid (Trolox) served as a standard for AO determination, enabling the description of obtained values as mg/g TEAC (Trolox Equivalent Antioxidant Capacity). All data were calculated on dry matter (DM), and measurements were performed using a JASCO V-530 spectrophotometer (Easton, MD, USA), with characteristic absorbance values set for each test and all samples measured in triplicate [4].

### 2.5. UPLC-ESI-QqQ-MS/MS Analysis

Sample extracts were diluted 1:50 (*v*:*v*) with 50% aqueous methanol acidified with 0.1% formic acid and then analyzed through an ACQUITY I-Class UPLC^TM^ separation system coupled to a Xevo TQ XS triple quadrupole mass spectrometer (Waters, Milford, MA, USA) equipped with an electrospray ionization source (ESI). Chromatographic separation was performed using a reversed-phase C18 ACQUITY UPLC HSS T3 column (2.1 × 100 mm, 1.8 µm particle size, Waters, Milford, MA, USA). For UPLC, water (eluent A) and acetonitrile (eluent B), both acidified with 0.01% formic acid, were used as mobile phases. The gradient started with 1% B, maintaining isocratic conditions for 0.5 min, followed by an increase to 15% B at 3 min, then to 50% B over 3 min, and finally to 95% B over 3 min. This 95% B condition was maintained for 1 min, followed by 0.1 min to reach 100% B. The system remained at 100% B for 1 min before returning to the initial conditions (1% B) in 1 min. Isocratic conditions were maintained for 3 min to re-equilibrate the column, resulting in a total run time of 14 min. The flow rate was set to 0.4 mL/min, the injection volume was 2 µL, and the column temperature was maintained at 40 °C. The MS operated in negative ionization mode with a desolvation temperature of 600 °C. The source temperature was set to 150 °C, and the source voltage was 2.3 kV. The compounds were monitored in multiple reaction monitoring (MRM) mode, with up to four molecular transitions used to qualify and quantify phenolic and other compounds. The system was controlled by MassLynx 4.2 software (Waters, Milford, MA, USA), and the data were processed using TargetLynx XS 4.1.1.0 software (Waters, Milford, MA, USA).

Metabolite identification was carried out by comparison of the retention time with analytical standards and/or MS/MS fragmentation patterns. Quantification was performed with calibration curves of standards, when available. When not available, metabolites were quantified with the most structurally similar compound.

### 2.6. Statistical Analysis

Chemical analyses carried out on in vitro-derived hop plantlets (cv. Columbus and Magnum) generated data that underwent two-way analysis of variance based on the factors “Genotype” (G) and “Extraction Method” (EM). Mean separation was performed using Tukey’s test (*p* ≤ 0.05) with SYSTAT 13.

## 3. Results

### 3.1. Total (Poly)Phenol Content and Antioxidant Activity

Chemical analysis of extracts obtained from in vitro-derived hop plantlets was conducted using several assays, and the results are presented in Table 1. The Folin–Ciocalteu test was used to quantify the TPC of in vitro-derived plantlets. Statistically significant differences were observed for the factor “Genotype” and the interaction between factors “Genotype” and “Extraction Method”. Regarding the “Genotype” factor, the samples of Columbus exhibited TPC statistically higher than that of Magnum (on the average, 5.92 ± 0.28 mg GAE/g vs. 5.62 ± 0.18 mg GAE/g, respectively). However, the values obtained from the interaction between the factors “Genotype” and “Extraction Method” were statistically higher for Columbus when extracted with ultrasound (6.10 ± 0.10 mg GAE/g vs. 5.53 ± 0.14 mg GAE/g, respectively). No statistically significant difference was observed in the analysis of extracts for both genotypes using the shaker extraction method (Table 1).

The DPPH^•^ test revealed statistically significant differences only for the “Genotype” factor. Specifically, Columbus showed, regardless of the extraction method used, antioxidant activity values higher than those of Magnum (on average, 4.21 ± 0.22 mg TEAC/mL vs. 3.93 ± 0.18 mg TEAC/mL, respectively). The extracts obtained from in vitro-derived hop plantlets were also evaluated using the ABTS^+^ test. The results showed no statistically significant differences between the cultivars (on average, Columbus 8.33 ± 0.41 mg TEAC/mL vs. Magnum 8.03 ± 0.32 mg TEAC/mL), nor for the “Extraction Method” factor. Finally, the FRAP assay revealed statistically significant differences only for the “Extraction Method” factor, irrespective of the genotype. Ultrasound-assisted extraction yielded, on average, a value of 6.78 ± 0.13 mg TEAC/mL, which was statistically higher than the corresponding value of 6.60 ± 0.06 mg TEAC/mL obtained through shaker extraction.

### 3.2. Characterization of Extracts from In Vitro-Derived Plantlets of Hop Genotypes through UPLC-ESI-QqQ-MS/MS

**Figure 2:**
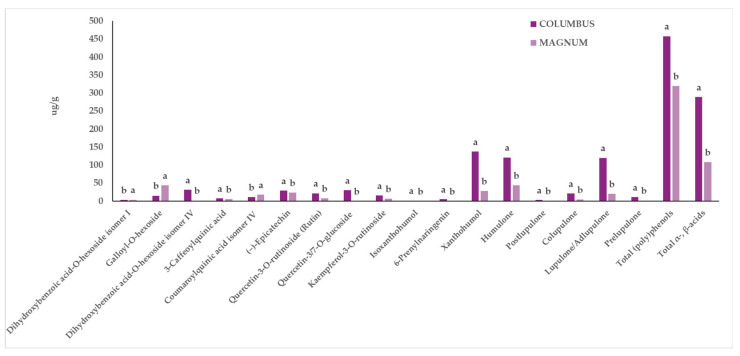
Influence of the “Genotype” factor on the phytochemical profile of in vitro-derived hop plantlets. Two-way ANOVA, Tukey’s test, *p* ≤ 0.05. Per each compound, different letters indicate statistically different values. The reported values were calculated per gram of dried plant material.

Extracts from two in vitro-derived plantlets of hop genotypes, Columbus and Magnum, were further investigated through UPLC-ESI-QqQ-MS/MS to assess their qualitative and quantitative profile in terms of phenolics and other phytochemical compounds. A total of 32 compounds belonging to the main families of phytochemicals characteristic of hop were identified, and 26 of them were quantified. These compounds included (poly)phenols (flavan-3-ols, flavonols, prenylflavonoids, hydroxybenzoic acids, and hydroxycinnamic acids) as well as α- and β-acids (Table 2). Statistical analysis revealed that, in general, the two considered factors (“G” and “EM”) did not interact, except in the case of sinapic acid acyl-hexoside. Statistically significant differences were reported for both “G” or “EM” factors. In general, the Columbus genotype showed higher amounts of most of the individual phytochemicals, as well as of total phenolics and total α- and β-acids, compared to those in Magnum (Figure 2, Table 3), regardless of the extraction method used. 

Specifically, Columbus was characterized by higher amounts of 3-caffeoylquinic acid, (−)-epicatechin, dihydroxybenzoic acid-*O*-hexoside (isomer IV), quercetin-3/7-*O*-glucoside (which is the sum of the two isomers coeluting, quercetin-3-*O*-glucoside and quercetin-7-*O*-glucoside), quercetin-3-*O*-rutinoside, kaempferol-3-*O*-rutinoside, isoxanthohumol, xanthohumol, 6-prenylnaringenin, and α- and β-acids (colupulone, lupulone/adlupulone, postlupulone, prelupulone, and humulone) (Figure 2). On the other hand, Magnum exhibited statistically significantly higher content of coumaroylquinic acid isomer IV, dihydroxybenzoic acid-*O*-hexoside (isomer I), and galloyl-*O*-hexoside (Figure 2, Table 3).

**Figure 3:**
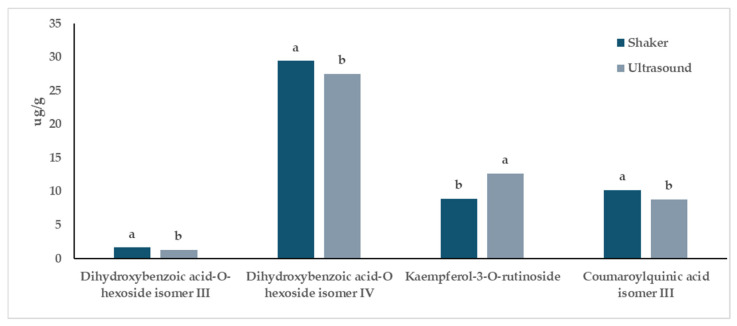
Influence of the “Extraction Method” factor on the phytochemical profile of in vitro-derived hop plantlets. Two-way ANOVA, Tukey’s test, *p* ≤ 0.05. Per each compound, different letters indicate statistically different values. The reported values were calculated per gram of dried plant material.

When considering the “Extraction Method” factor, a comparison between the two extraction methods, regardless of the cultivar considered, revealed differences for 6 out of 26 compounds (Figure 3, Table 3). Specifically, (−)-epicatechin and kaempferol-3-*O*-rutinoside were better extracted using sonication, while coumaroylquinic acid isomer III and dihydroxybenzoic acid hexoside isomers III and IV showed higher extraction efficiency when the shaker method was employed (Figure 3, Table 3). Sinapic acid acyl-hexoside was better extracted using sonication only for Magnum (Table 3).

## 4. Discussion

The bioactive compounds of hop plants are not only found in plants grown in the field, but also in plants cultivated in vitro [4,15]. The extraction of these valuable compounds from in vitro-derived hop plantlets can alleviate the problems associated with the high sensitivity of secondary metabolism of field-grown plants to environmental conditions [5,16]. Indeed, cultivating plantlets in vitro ensures controlled conditions that allow for standardization of types and concentrations of bioactive compounds that will provide a consistent product to stakeholders. Furthermore, as hop is a deciduous plant that loses its leaves at the end of summer, it can be difficult to guarantee the supply of vegetative biomass throughout the year, while resorting to in vitro techniques would allow for continuous production of micropropagated plants, which can be grown independently of seasonal changes [4].

In this study, hop plants of the cultivars Columbus and Magnum obtained in vitro were used as a matrix for the extraction of bioactive compounds. For the extraction, the protocol described by Carbone et al. [18] for hop cones and modified by Chiancone et al. [4] for in vitro-derived hop material was used, with an 80% ethanol solution as the solvent, which may be considered an environmentally friendly extraction method.

Extracts obtained from both cultivars were characterized for their TPC and antioxidant activity using different assays, including DPPH^•^, ABTS^+^, and FRAP. To the best of authors’ knowledge, this is the second study on (poly)phenol content and antioxidant activity of in vitro-derived hop plantlets; the first was that in which the chemical characterization was carried out considering separately the different parts of the plantlets (leaves and roots) [4]. The present research represents a step ahead of the study of Chiancone et al. [4]; in fact, two new genotypes were studied, and the obtained (poly)phenolic profile gave a larger amount of information. Given the lack of research on this subject, other than Chiancone et al. [4], the results obtained in this work were mainly compared with those of other studies on hop plants grown in the field.

With regard to TPC, the values obtained for in vitro-derived plant material were comparable to those found in hop leaves and cones [20,21], even if in some cases the extraction was conducted applying a solvent different from ethanol. Looking at the material obtained in vitro, the extracts obtained from Columbus plantlets considered in this study had a TPC comparable to that of Gianni but lower than that of Cascade, while the TPC of Magnum was lower than that of all considered genotypes [4].

Significant influence of the extraction method on the TPC of in vitro-derived plantlets was observed, with higher TPC when ultrasound was used. In previous work using the same extraction method, no differences were observed between the two genotypes considered, with an average TPC of 5.8 mg GAE/g, a value lower than that observed in this study for cv. Columbus but higher than that of Magnum [4]. 

Three different antioxidant capacity assays were performed. This is important to discriminate among compounds that absorb at similar wavelengths and may have different mechanisms, as recommended by Moon and Shibamoto [22]. The factor “Genotype” statistically influenced the results of the DPPH^•^ assay, and both genotypes showed values higher than those of the Cascade and Gianni genotypes reported by Chiancone et al. [4]. In general, the results obtained in vitro for the DPPH^•^ assay were significantly higher than those obtained from hop leaves harvested in the field [2,20].

The antioxidant activity of both cultivars was further evaluated using the ABTS^+^ assay and was not influenced by the cultivar, extraction method, or their interaction. This result is consistent with the findings of Chiancone et al. [4], which suggested that the lack of statistically significant differences may be attributed to the differing composition of the (poly)phenolic portion of these hop cultivars and/or to a different response of (poly)phenolic compounds when in contact with the radicals detected by this assay.

A final test to evaluate the antioxidant activity of the samples was conducted using the FRAP assay. A statistically significant interaction was observed for the “Extraction Method” factor, with ultrasonic extraction resulting in higher values compared to those of traditional methods such as shaking (67.85 mg TEAC/mL vs. 66.00 mg TEAC/mL, respectively). These results are consistent with those reported by Abid et al. [23].

Information about antioxidant activity is highly relevant, considering potential applications for the food industry. Extracts from in vitro-derived hop plantlets could be used as a natural antioxidant to enhance the shelf-life of certain products (such as biscuits, crackers, or other bakery products, for example) while meeting consumer preferences for natural ingredients and clean labels [24,25]. In the context of human health, their in vivo antioxidant activity remains uncertain. In fact, when ingested, bioactive compounds present in extracts, and especially phenolic compounds, are subjected to extensive metabolism that originates a plethora of metabolites that differ from the native molecules [26,27]. In this sense, it is difficult to ascertain if the newly originated metabolites have the same antioxidant activity of the parent compounds in vivo. Additionally, the molecular mechanisms by which phenolic metabolites may confer their potential health benefits do not rely mainly on a direct antioxidant effect, as thought in the past, but probably on diverse actions within intra- and inter-cellular signaling pathways (including regulation of nuclear transcription factors and fat metabolism, modulation of the synthesis of inflammatory mediators such as the cytokines tumor necrosis factor α, interleukin-1β, and interleukin-6) and on other indirect antioxidant activities protecting cells and tissues [27,28,29,30,31].

Regarding the phytochemical profile of the in vitro-derived hop plantlets, the results of this study were in line to those of Chiancone et al. [4], who characterized in vitro-derived hop leaves and roots of two hop plant types, the variety Cascade and the ecotype Gianni. In the present study, a higher number of compounds was identified (Table 2), and quantification (or semi-quantification) data were provided. 

The characterization carried out was in line with the (poly)phenolic and bitter-tasting organic acid profile of hop. Indeed, the phytochemical profiles of the in vitro-derived hop plantlets from both Columbus and Magnum cultivars were comparable to those reported for hops grown in open fields (cones and leaves) [32,33,34,35]. However, when considering the quantitative composition, the Columbus cultivar exhibited higher levels of most identified phytochemicals compared to those in the Magnum cultivar, regardless of the extraction method applied, consistent with the results obtained from the TPC and DPPH^•^ assays. Even if at levels markedly lower due to the starting material used, in vitro-derived plantlets of Columbus presented an amount of total phenolics higher than that in Magnum, as reported by Mongelli et al. [35] for cones of the same cultivars. 

Xanthohumol, humulone, and lupulone/adlupulone (the sum of the two isomers) were the most abundant compounds found in the extracts of Columbus samples (Figure 2). Additionally, besides the characteristic hop phytochemicals, the Columbus variety also presented significantly higher content of certain (poly)phenols, such as 3-caffeoylquinic acid, (−)-epicatechin, an isomer of dihydroxybenzoic acid hexoside, quercetin-3/7-*O*-glucoside, quercetin-3-*O*-rutinoside, and kaempferol-3-*O*-rutinoside (Figure 2).

Regarding the extraction methods applied, their efficiency in extracting phytochemicals from both Columbus and Magnum in vitro-derived hop plantlets was similar. Indeed, the comparison between the two extraction methods revealed differences for only 6 out of 26 compounds (Figure 3, Table 3). Coumaroylquinic acid isomer III and dihydroxybenzoic acid hexoside isomers III and IV were better extracted using the shaker, while (−)-epicatechin, kaempferol-3-*O*-rutinoside, and sinapic acid acyl-hexoside (this latter compound only for Magnum) were better extracted using sonication (Figure 3, Table 3). Nevertheless, from a biological point of view, these differences were negligible and should not drive commercial extraction strategies. On the other hand, these results partly contrasted with those obtained from TPC and FRAP assays, which reported higher (poly)phenolic content and antioxidant activity for samples extracted using ultrasound, regardless of the cultivar. These discrepancies might be attributed to the well-known limitations of these assays [36,37]. Furthermore, recent studies conducted on hop pellet samples have shown that the extraction technique, and in particular extractions conducted with a shaker and/or ultrasonic bath, lead to different results depending on the type of solvent that is used [38]. Therefore, concerning the material considered in the present study, considering the higher speed associated with the application of ultrasonic extraction, this method might be preferred for future studies.

## 5. Conclusions

Hop plants have been demonstrated to be a wealth source of bioactive compounds, but open field-grown plants cannot satisfy the increasing demand of these compounds. In vitro cultures, allowing for continuous, standardized plant production, represent a valid support in order to guarantee to stakeholders a constant supply of plant material to be used for reproducible bioactive compound extraction.

The cultivars Columbus and Magnum were utilized as a matrix to extract bioactive compounds. The TPC results indicated that the richness in the phytochemical profile of in vitro-derived hop plantlets was similar between the Columbus and Magnum cultivars and comparable to that reported for hop plants grown in open fields. However, while performing a quali–quantitative profiling, the Columbus cultivar exhibited higher amounts of most identified phytochemicals compared to those in the Magnum cultivar, including prenylated flavonoids (isoxanthohumol, xanthohumol, 6-prenylnaringenin), α-acids (humulone), and β-acids (colupulone, lupulone/adlupulone, postlupulone, prelupulone). 

Regarding the extraction methods applied, their efficiency in extracting phytochemicals from in vitro-derived plantlets was similar. However, ultrasonic extraction resulted in higher TPC and antioxidant activity compared to those with traditional methods such as shaking. Despite some discrepancies observed among different assays tested, the ultrasonic method, which allows reducing the extraction time, will be selected for future studies.

In conclusion, this study provides valuable insights into the phytochemical profile and antioxidant activity of in vitro-derived hop plantlets from Columbus and Magnum cultivars. These findings contribute to a better understanding of the potential benefits of hop-derived products and may inform future research in this area.

## Figures and Tables

**Table 1:** Total phenolic content (TPC) and antioxidant activity, measured by DPPH^•^, ABTS^+^ and FRAP assays, of extracts from hop in vitro-derived plantlets of two genotypes, “Columbus” and “Magnum” (mean ± SD).

Genotype	Extraction Method	TPC		DPPH^•^		ABTS^+^		FRAP	
		mg GAE/g	±SD	mg TEAC/mL	±SD	mg TEAC/mL	±SD	mg TEAC/mL	±SD
**Columbus**	**Untrasound**	6.10	0.10	4.30	0.10	8.26	0.32	6.84	0.14
	**Shaker**	5.75	0.29	4.12	0.26	8.41	0.47	6.62	0.07
**Magnum**	**Untrasound**	5.53	0.14	3.88	0.10	8.24	0.19	6.73	0.09
	**Shaker**	5.71	0.18	3.99	0.21	7.81	0.28	6.58	0.05
Statistical analysis of factors									
		*p*		*p*		*p*		*p*	
**GENOTYPE (G)**		**0.018**		**0.025**		0.135		0.220	
**EXTRACTION METHOD (EM)**		0.431		0.754		0.472		**0.006**	
**G×EM**		**0.033**		0.197		0.156		0.508	

Two-way analysis of variance (ANOVA), Tukey’s test (*p* ≤ 0.05). Bold *p* values were statistically significant (*p ≤* 0.05). Abbreviations: US, ultrasound; SK, shaker; G, genotype; EM, extraction method; SD, standard deviation.

**Table 2:** Mass spectral characteristic of hop phytochemicals identified and quantified in extracts from two in vitro-derived plantlets of hop genotypes, Columbus and Magnum. Bold highlights the compounds identified by means of authentic standards.

Family	Compound	RT (min)	Parent Ion [M-H]^−^ (*m*/*z*)	Product Ions				Standard Used for Quantification
				Quantifier (*m*/*z*)	Qualifier(s) (*m*/*z*)			
Hydroxybenzoic acids	**3,4,5-Trihydroxybenzoic acid (Gallic acid)**	2.36	169	125	97			<LOQ
	Dihydroxybenzoic acid- *O*-hexoside isomer I	2.60	315	153	109	152	108	3,4-Dihydroxybenzoic acid
	Dihydroxybenzoic acid- *O*-hexoside isomer II	2.65	315	152	153	109	108	3,4-Dihydroxybenzoic acid
	Dihydroxybenzoic acid- *O*-hexoside isomer III	2.83	315	153	109	152	108	3,4-Dihydroxybenzoic acid
	Galloyl-*O*-hexoside	2.87	331	169	125			3,4,5-Trihydroxybenzoic acid
	Dihydroxybenzoic acid- *O*-hexoside isomer IV	3.17	315	153	109	152	108	3,4-Dihydroxybenzoic acid
	3,4-Dihydroxybenzoic acid (Protocatechuic acid)	3.19	153	109	81			<LOQ
Hydroxycinnamic acids	3-Caffeoylquinic acid	3.30	353	191	179	135		3-Caffeoylquinic acid
	Coumaroylquinic acid isomer I	3.77	337	191	173			3-Caffeoylquinic acid
	Coumaroylquinic acid isomer II	3.82	337	191	173			3-Caffeoylquinic acid
	5-Caffeoylquinic acid	3.84	353	191	179	135		<LOQ
	4-Caffeoylquinic acid	3.93	353	179	191	135		<LOQ
	Sinapic acid acyl-hexoside	3.93	385	223	205	190		Sinapic acid acyl- glucoside
Flavan-3-ols	(+)-Catechin	3.97	289	245	109	203		(+)-Catechin
Hydroxycinnamic acids	Coumaroylquinic acid isomer III	4.29	337	173	191			4-Caffeoylquinic acid
	Sinapic acid acyl-glucoside	4.29	385	205	223	190		<LOQ
	Coumaroylquinic acid isomer IV	4.34	337	191	173			5-Caffeoylquinic acid
Flavan-3-ols	(−)-Epicatechin	4.37	289	245	109	203		(−)-Epicatechin
Flavonols	Quercetin 3-*O*-rutinoside (Rutin)	4.73	609	300	271	301	151	Kaempferol 3-rutinoside
	Quercetin 3/7-*O*-glucoside (Sum of Quercetin 3-*O*-glucoside and Quercetin-7-*O*-glucoside)	4.86	463	300	271	255		Quercetin 3/7-glucoside (Sum of Quercetin 3-glucoside and Quercetin-7-glucoside)
	Kaempferol-3-*O*-rutinoside	4.95	593	285	255	227		Kaempferol 3-rutinoside
Prenylflavonoids	Isoxanthohumol	6.88	353	119	233			Isoxanthohumol
	8-Prenylnaringenin	7.51	339	219	95	237		<LOQ
Iso-α-acids	Isohumulone	7.54	361	221	292	249		Humulone
Prenylflavonoids	6-Prenylnaringenin	8.04	339	219	119	133		6-Prenylnaringenin
	Xanthohumol	8.29	353	119	233	175		Xanthohumol
α-acids	Cohumulone	9.42	347	278	235	223		Humulone
	Humulone	9.65	361	292	249	221		Humulone
β-acids	Postlupulone	9.91	385	273				Humulone
	Colupulone	10.18	399	287	330			Humulone
	Lupulone/Adlupulone	10.42	413	301	289			Humulone
	Prelupulone	10.78	427	315	358			Humulone

RT, retention time. <LOQ means that the compound was identified but not quantified because it was under the limit of quantification.

**Table 3:** Influence of the genotype and extraction method on the phytochemical composition of in vitro-derived hop plantlets.

	Columbus				Magnum				Factors		
Compound	US		SK		US		SK		G	EM	G × EM
	µg/g	±SD	µg/g	±SD	µg/g	±SD	µg/g	±SD	*p*	*p*	*p*
Dihydroxybenzoic acid-*O*-hexoside isomer I	3.02	±0.06	2.93	±0.42	4.04	±0.03	3.76	±0.03	**0.012**	0.436	0.676
Dihydroxybenzoic acid-*O*-hexoside isomer II	40.79	±6.57	39.72	±0.13	27.59	±2.84	42.47	±2.74	0.244	0.146	0.106
Dihydroxybenzoic acid-*O*-hexoside isomer III	1.44	±0.04	1.61	±0.08	1.18	±0.13	1.61	±0.14	0.307	**0.050**	0.296
Galloyl-*O*-hexoside	14.26	±0.23	16.12	±0.72	47.78	±3.28	40.61	±0.65	**0.000**	0.197	0.058
Dihydroxybenzoic acid-*O*-hexoside isomer IV	29.29	±0.19	33.14	±1.19	21.05	±0.75	25.71	±1.56	**0.002**	**0.016**	0.722
3-Caffeoylquinic acid	8.07	±1.28	7.25	±0.85	5.00	±0.28	5.60	±0.41	**0.043**	0.899	0.426
Coumaroylquinic acid isomer I	2.46	±0.35	2.69	±0.07	2.72	±0.08	2.71	±0.09	0.498	0.601	0.571
Coumaroylquinic acid isomer II	6.30	±0.93	6.85	±0.70	6.17	±0.26	8.85	±0.08	0.193	0.053	0.150
Sinapic Acid Acyl-hexoside	45.22	±1.12	47.02	±2.40	61.03	±0.83	41.58	±3.67	0.088	**0.019**	**0.01**
(+)-Catechin	39.24	±1.38	44.73	±6.19	82.64	±14.75	44.03	±14.99	0.124	0.206	0.115
Coumaroylquinic acid isomer III	8.55	±0.37	9.36	±0.41	9.10	±0.33	11.00	±0.56	0.062	**0.034**	0.271
Coumaroylquinic acid isomer IV	11.08	±1.03	10.57	±0.26	17.14	±0.20	17.82	±0.57	**0.000**	0.898	0.389
(−)-Epicatechin	33.47	±2.99	24.10	±0.29	13.09	±0.89	9.16	±1.80	**0.001**	**0.021**	0.206
Quercetin-3-*O*-rutinoside (Rutin)	22.12	±2.27	21.68	±3.01	10.36	±0.27	5.82	±0.53	**0.002**	0.262	0.342
Quercetin-3/7-*O*-glucoside (Sum of Quercetin3-*O*-Glucoside and Quercetin-7-*O*-glucoside)	31.36	±1.20	30.06	±5.94	0.00	±0.00	0.00	±0.00	**0.000**	0.776	0.776
Kaempferol-3-*O*-rutinoside	16.79	±1.31	13.70	±2.27	8.47	±0.57	4.06	±0.34	**0.003**	**0.050**	0.651
Isoxanthohumol	1.67	±0.01	1.61	±0.09	0.00	±0.00	0.00	±0.00	**0.000**	0.574	0.574
Isohumulone	0.56	±0.01	0.56	±0.00	0.55	±0.01	0.57	±0.00	0.719	0.388	0.138
6-Prenylnaringenin	5.57	±0.19	5.17	±0.28	0.00	±0.00	0.00	±0.00	**0.000**	0.300	0.300
Xanthohumol	141.99	±7.36	133.63	±7.05	22.09	±1.43	34.28	±5.70	**0.000**	0.761	0.156
Cohumulone	12.50	±1.05	13.29	±1.07	7.70	±0.76	15.24	±4.02	0.548	0.129	0.196
Humulone	120.54	±15.25	121.97	±3.54	48.15	±7.32	92.95	±22.48	**0.023**	0.178	0.201
Postlupulone	2.99	±0.32	2.72	±0.18	0.47	±0.01	0.76	±0.16	**0.000**	0.971	0.237
Colupulone	22.39	±3.69	19.91	±0.55	2.99	±0.03	5.55	±1.37	**0.001**	0.985	0.273
Lupulone/Adlupulone	131.18	±20.23	108.85	±3.51	13.71	±0.11	26.45	±5.35	**0.001**	0.675	0.174
Prelupulone	11.98	±2.61	10.13	±0.09	1.07	±0.02	2.01	±0.49	**0.002**	0.750	0.352
Total (poly)phenols	463.24	±17.70	452.5	±21.1	340.01	±14.98	299.63	±12.52	**0.000**	0.089	0.282
Total α- and β-acids	301.57	±61.02	276.85	±12.38	74.08	±11.24	142.96	±47.90	**0.000**	0.475	0.171

Two-way analysis of variance (ANOVA), Tukey’s test (*p* ≤ 0.05). Bold *p* values were statistically significant (*p* ≤ 0.05). Abbreviations: US, ultrasound; SK, shaker; G, genotype; EM, extraction method; SD, standard deviation.

## Data Availability

The original contributions presented in the study are included in the article, further inquiries can be directed to the corresponding author.
