# Correction: Leto et al. Evaluation of In Vitro-Derived Hop Plantlets, cv. Columbus and Magnum, as Potential Source of Bioactive Compounds. *Antioxidants* 2024, *13*, 909

**DOI:** 10.3390/antiox14080922

**Published:** 2025-07-29

**Authors:** Leandra Leto, Claudia Favari, Anna Agosti, Lorenzo Del Vecchio, Andrea Di Fazio, Letizia Bresciani, Pedro Mena, Valeria Guarrasi, Martina Cirlini, Benedetta Chiancone

**Affiliations:** 1 Department of Food and Drug, University of Parma, Viale Parco Area delle Scienze 27/A, 43124 Parma, Italy; leandra.leto@unipr.it (L.L.); claudia.favari@unipr.it (C.F.); anna.agosti@unipr.it (A.A.); lorenzo.delvecchio@unipr.it (L.D.V.); andrea.difazio@studenti.unipr.it (A.D.F.); letizia.bresciani@unipr.it (L.B.); pedro.mena@unipr.it (P.M.); martina.cirlini@unipr.it (M.C.); 2 Institute of Biophysics, National Research Council (CNR), Via Ugo La Malfa 153, 90146 Palermo, Italy; valeria.guarrasi@ibf.cnr.it

In the original publication [1], there was a mistake in Table 1 as published. The necessary correction relates to incorrect values in the antioxidant activity assays (DPPH, ABTS, FRAP) due to an error in the conversion factor. Specifically, all values should be divided by 10. The corrected Table 1 appears below. 

Due to mistakes in the dataset, values reported in the text needed to be corrected. A correction has been made to Section 3.1 Total (Poly)Phenol Content and Antioxidant Activity, paragraph 2:

The DPPH^•^ test revealed statistically significant differences only for the “Genotype” factor. Specifically, Columbus showed, regardless of the extraction method used, antioxidant activity values higher than those of Magnum (on average, 4.21 ± 0.22 mg TEAC/mL vs. 3.93 ± 0.18 mg TEAC/mL, respectively). The extracts obtained from in vitro-derived hop plantlets were also evaluated using the ABTS^+^ test. The results showed no statistically significant differences between the cultivars (on average, Columbus 8.33 ± 0.41 mg TEAC/mL vs. Magnum 8.03 ± 0.32 mg TEAC/mL), nor for the “Extraction Method” factor. Finally, the FRAP assay revealed statistically significant differences only for the “Extraction Method” factor, irrespective of the genotype. Ultrasound-assisted extraction yielded, on average, a value of 6.78 ± 0.13 mg TEAC/mL, which was statistically higher than the corresponding value of 6.60 ± 0.06 mg TEAC/mL obtained through shaker extraction.

The authors state that the scientific conclusions are unaffected. This correction was approved by the Academic Editor. The original publication has also been updated.

**Table 1:** Total phenolic content (TPC) and antioxidant activity, measured by DPPH^•^, ABTS^+^ and FRAP assays, of extracts from hop in vitro-derived plantlets of two genotypes, “Columbus” and “Magnum” (mean ± SD).

Genotype	Extraction Method	TPC		DPPH^•^		ABTS^+^		FRAP	
		mg GAE/g	±SD	mg TEAC/mL	±SD	mg TEAC/mL	±SD	mg TEAC/mL	±SD
**Columbus**	**Untrasound**	6.10	0.10	4.30	0.10	8.26	0.32	6.84	0.14
	**Shaker**	5.75	0.29	4.12	0.26	8.41	0.47	6.62	0.07
**Magnum**	**Untrasound**	5.53	0.14	3.88	0.10	8.24	0.19	6.73	0.09
	**Shaker**	5.71	0.18	3.99	0.21	7.81	0.28	6.58	0.05
Statistical analysis of factors									
		*p*		*p*		*p*		*p*	
**GENOTYPE (G)**		**0.018**		**0.025**		0.135		0.220	
**EXTRACTION METHOD (EM)**		0.431		0.754		0.472		**0.006**	
**G×EM**		**0.033**		0.197		0.156		0.508	

Two-way analysis of variance (ANOVA), Tukey’s test (*p* ≤ 0.05). Bold *p* values were statistically significant (*p ≤* 0.05). Abbreviations: US, ultrasound; SK, shaker; G, genotype; EM, extraction method; SD, standard deviation.
